# Silent saboteur: parvovirus B19 (B19V) and its impact on refractory post-transplant anemia

**DOI:** 10.3389/fmed.2025.1686727

**Published:** 2025-12-12

**Authors:** Mythri Shankar, Varalaxmi Shetty, Dwarak Sampath Kumar, Sreedhara C. G.

**Affiliations:** Department of Nephrology, Institute of Nephro Urology, Bengaluru, India

**Keywords:** parvovirus B19, kidney transplantation, refractory anemia, immunosuppression reduction, intravenous immunoglobulin

## Abstract

**Introduction:**

Anemia is a common and multifactorial complication following kidney transplantation, significantly impacting patient morbidity and graft survival. Parvovirus B19 (B19V), a DNA virus targeting erythroid progenitor cells, is an increasingly recognized cause of refractory anemia in kidney transplant recipients (KTRs), but data from Indian populations remain sparse.

**Methods:**

This single-center case series evaluated 10 KTRs diagnosed with B19V infection at the Institute of Nephro Urology, Bengaluru, from January 2021 to February 2025. Inclusion criteria encompassed KTRs with persistent anemia unresponsive to routine correction, confirmed by positive polymerase chain reaction (PCR) for B19V and/or characteristic bone marrow findings. Clinical parameters, laboratory data, immunosuppression details, treatment, and outcomes were collected prospectively over a minimum 3-months follow-up. Treatment included reduction of immunosuppression, with adjunct intravenous immunoglobulin (IVIg) for non-responders, and use of erythropoiesis-stimulating agent Desidustat as adjunct treatment in all cases.

**Results:**

Among 117 transplant recipients, 8.5% developed B19V infection, with a median infection onset of 6 weeks (range 2–462 weeks). All (100%) presented with fatigue and hypoproliferative anemia (reticulocyte count < 1%). 30% patients also had leukopenia and thrombocytopenia. 60% patients required IVIg post-reduction of immunosupression, achieving hematologic recovery within 4 weeks. Eight patients needed packed red blood cell transfusions (range 1–8 units). Two patients succumbed to severe infections unrelated to B19V. Statistical analysis confirmed significant decline in hemoglobin (mean reduction 3.72 ±1.22 g/dL, *p* < 0.001).

**Conclusion:**

Parvovirus B19 is an important yet treatable cause of refractory anemia in KTRs. Early diagnosis by PCR and tailored management incorporating reduction in immunosuppression and IVIg can result in favorable outcomes. Routine surveillance is not warranted, but targeted testing should be considered in refractory anemia cases, particularly in resource-limited settings. Further studies are needed to optimize therapeutic strategies and validate novel treatments such as Desidustat.

## Introduction

Anemia is a frequent and multifactorial complication following kidney transplantation that significantly impacts patient morbidity, graft function, and survival. Up to 90% of kidney transplant recipients (KTRs) develop anemia in the early post-transplant period, with 35%–45% continuing to experience anemia at 1 year despite stable graft function. Multiple factors contribute to post-transplant anemia, including iron deficiency, erythropoietin insufficiency, immunosuppressive medication effects, graft dysfunction or rejection, infections, malignancy, acute and chronic bleeding. Opportunistic viral infections such as cytomegalovirus (CMV), Epstein-Barr virus (EBV), BK polyomavirus, and B19V are recognized contributors to anemia in this population (1, 2).

Parvovirus B19 is a small, single-stranded DNA virus of the Parvoviridae family, which selectively infects erythroid progenitor cells by binding to the P antigen receptor on their surface (3–5).

Parvovirus B19 is a globally distributed DNA virus with a reported IgG seroprevalence of 60%–80% in adults, but around 20%–40% of transplant candidates remain seronegative and thus at risk for primary infection. In kidney transplant recipients, B19V infection occurs most commonly within the first 3 months post-transplant, with reported prevalence rates ranging from 1% to 15%, and is often associated with intensified immunosuppression. The primary clinical manifestation in this setting is severe, persistent anemia that may manifest as pure red cell aplasia due to direct cytotoxic effects on erythroid progenitors, with associated symptoms including fatigue, weakness, and in some cases, leukopenia and thrombocytopenia. Classic rash or arthropathy–hallmarks of B19V in immunocompetent individuals–are uncommon in transplant populations due to blunted immune responses, making diagnosis challenging and reliant on nucleic acid testing for B19V DNA. Awareness of these features is essential for prompt recognition and management to prevent significant morbidity in transplant patients (3–5).

Diagnosis is most reliably established by detecting B19V DNA through polymerase chain reaction (PCR), as antibody responses may be diminished or absent under immunosuppression. Bone marrow examination, though not always performed, may reveal giant pronormoblasts with inclusion bodies and markedly decreased erythropoiesis (6, 7).

An emerging body of evidence suggests that erythropoietin (EPO) therapy may have complex interactions with parvovirus B19 (B19V) infection in transplant recipients. Recent studies indicate that EPO can enhance B19V replication, which may worsen the severity or duration of viremia in affected patients. Furthermore, these studies highlight that EPO administration might diminish the therapeutic efficacy of intravenous immunoglobulin (IVIg), a cornerstone of B19V infection treatment in post-transplant anemia. As a result, careful consideration and monitoring of EPO use is warranted in transplant recipients with proven or suspected B19V infection to optimize hematologic recovery and improve patient outcomes (8).

Despite increasing awareness globally, data from India on B19V infection-related anemia in KTRs remain limited, with few reports discussing prevalence, clinical course, treatment, and outcomes (2, 9). The mainstay of treatment involves reduction of immunosuppression to enhance the host’s immune clearance of the virus, supplemented by intravenous immunoglobulin (IVIg) therapy in refractory cases. However, optimal dosing and duration of IVIg remain unclear, and financial constraints may limit its use in resource-poor settings. Emerging therapies such as hypoxia-inducible factor prolyl-hydroxylase inhibitors (HIF-PHIs) like Desidustat, which stimulate endogenous erythropoietin production, may serve as useful adjuncts but require further evaluation (10).

This case series reports clinical, laboratory, treatment, and outcome data of ten kidney transplant recipients with B19V-associated anemia from a tertiary referral center in India. These findings aim to improve recognition and management of this underdiagnosed cause of refractory anemia in transplant populations.

## Materials and methods

This single-center case series was conducted at the Institute of Nephro Urology, Bengaluru, evaluating kidney transplant recipients (KTRs) who developed unexplained, persistent anemia following transplantation between January 2021 and February 2025.

### Patient selection

Inclusion criteria comprised KTRs presenting with refractory anemia characterized by hemoglobin levels below 13 g/dL in males and below 12 g/dL in females, with reticulocyte counts less than 1%, and in whom common reversible causes of anemia had been excluded. Patients were further evaluated for B19V infection based on clinical suspicion of persistent hypoproliferative anemia (1, 9).

### Diagnostic criteria

Diagnosis of B19V infection was established by: Positive qualitative polymerase chain reaction (PCR) for B19V DNA in peripheral blood samples, with or without (1, 4).

Bone marrow examination demonstrating decreased erythroid lineage and the presence of giant pronormoblasts with viral inclusions suggestive of parvoviral infection (5).

Polymerase chain reaction for B19V DNA in blood was the defining diagnostic criterion. Bone marrow findings were supportive but not definitive.

Additional hematological parameters assessed included leukopenia (white blood cell count < 4000 cells/μL) and thrombocytopenia (platelet count < 150,000/μL) (9).

We used Commercial kits - the Human Parvovirus B19 Real Time PCR Kit, which are validated to detect all three major B19V genotypes, using fluorescent probes to monitor amplification in real time. The sensitivity of these assays is high, detecting as few as 10 copies of viral genome per reaction (11). Viral load quantification was performed.

### Data collection and follow-up

Clinical presentation, laboratory data, immunosuppressive regimens, induction therapies, and treatment modifications were recorded. Patients were followed for at least 3 months post-diagnosis to monitor hemoglobin trends, transfusion requirements, graft function, and overall outcomes.

### Treatment protocol

**FIGURE 1:**
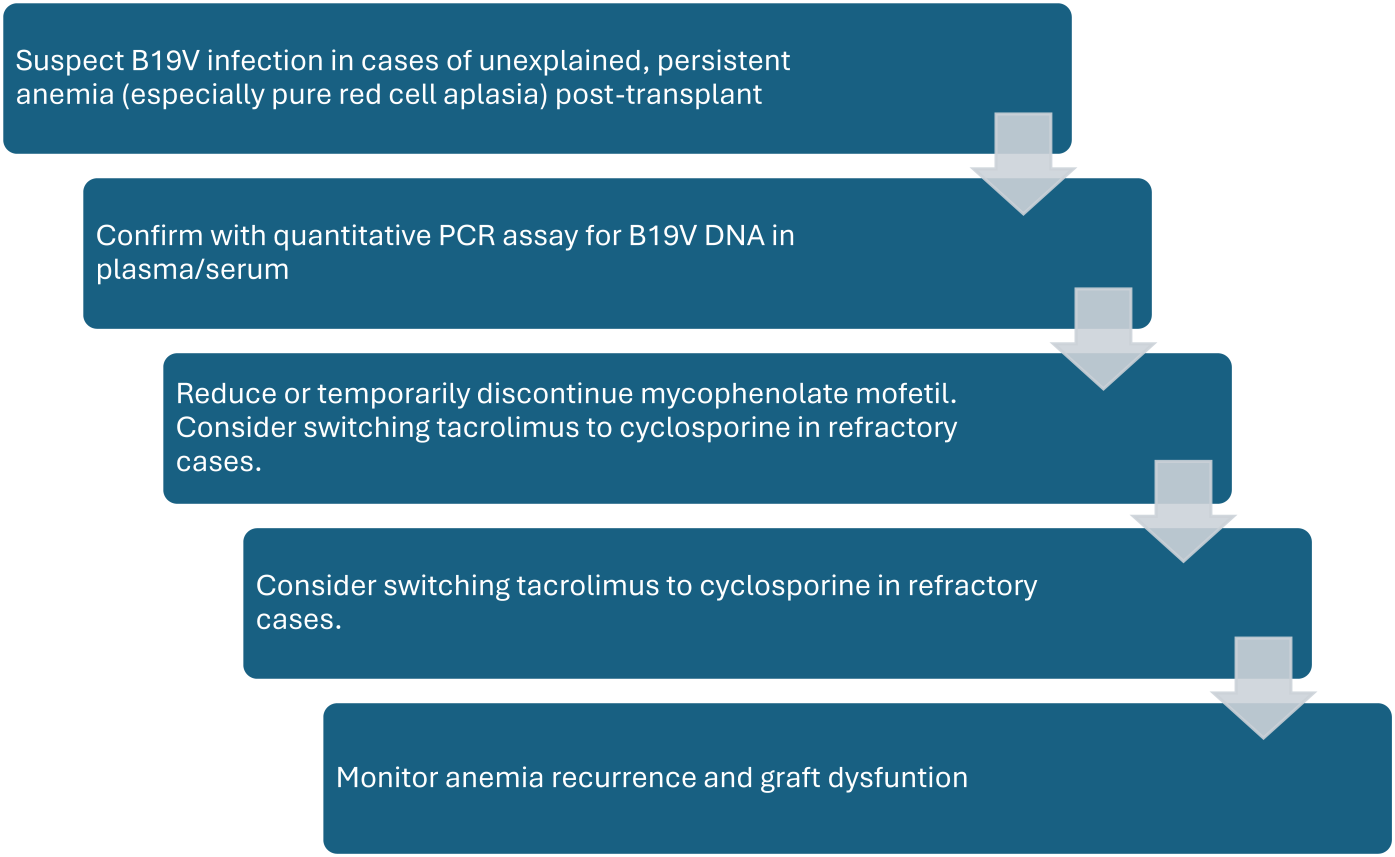
Treatment protocol. All patients received Desidustat as adjunct therapy and supportive packed red blood cell transfusion if Hb < 7 g/dl.

Initial management involved correction of common reversible causes of anemia, such as iron, vitamin B12, and folate deficiencies. Definitive therapy consisted primarily of reduction in immunosuppression (RIS), particularly tapering or withholding mycophenolate mofetil (MMF), tailored to individual clinical status. If still no response, tacrolimus was changed to cyclosporine (7, 12) (Figure 1).

Patients who failed to show hematological improvement with RIS alone within 7–10 days received adjunct intravenous immunoglobulin (IVIg) therapy at a dose of 400 mg/kg daily for five consecutive days (8).

Packed red blood cell transfusions were administered for severe symptomatic anemia, defined as hemoglobin below 7 g/dL.

All patients received Desidustat as adjunct therapy, a hypoxia-inducible factor prolyl hydroxylase inhibitor (HIF-PHI) approved in India, to stimulate endogenous erythropoietin production (9).

### Response assessment

Treatment response was defined by a sustained increase in hemoglobin level, evidenced by two consecutive hemoglobin measurements taken at least 1 week apart demonstrating an upward trend (8).

## Results

Between January 2021 and February 2025, a total of 117 kidney transplant recipients (KTRs) were evaluated at our center. Among these, 10 patients (8.5%) were diagnosed with B19V infection-associated refractory anemia based on clinical, laboratory, and molecular criteria.

### Patient demographics and clinical characteristics

The cohort comprised 7 (70%) males and 3 (30%) females with a median age of 38 years (range 19–58 years). The median time from transplantation to diagnosis of B19V infection was 6 weeks (range 2–462 weeks). All patients presented with fatigue and hypoproliferative anemia, characterized by reticulocyte counts consistently below 1%, indicative of erythropoietic suppression (Table 1).

**TABLE 1:** Clinical and laboratory parameters of the cases.

Parameter	Normal values	Patient 1	Patient 2	Patient 3	Patient 4	Patient 5	Patient 6	Patient 7	Patient 8	Patient 9	Patient 10
Age in years		38	44	45	58	37	49	29	19	33	35
Sex		F	M	M	M	M	M	F	F	M	M
Type of transplant		LRRATX	LRRATX	DDRATX	DDRATX	DDRATX	LRRATX	LRRATX	DDRATX	DDRATX	LRRATX
Induction		No induction	ATLG (3.5 mg/kg single dose)	ATLG (3.5 mg/kg single dose)	ATLG (3.5 mg/kg single dose)	ATLG (3.5 mg/kg single dose)	No induction	No induction	ATLG (3.5 mg/kg single dose)	ATLG (3.5 mg/kg single dose)	No induction
Rejection episode prior to parvo detection		Yes	No	No	No	No	No	No	No	No	Yes
Time of infection from transplant (weeks)		3	8	4	2	9	256	49	4	4	462
Presentation		Fatigue	Fatigue	Fatigue	Fatigue	Fatigue	Fatigue	Fatigue	Fatigue	Fatigue	Fatigue
Method of diagnosis		PCR	PCR	PCR	PCR	PCR	PCR	PCR	PCR	PCR	PCR
Baseline Hb (prior to parvovirus infection) (mg/dl)		9 g/dl	10 g/dl	10 g/dl	11 g/dl	9 g/dl	10 g/dl	10 g/dl	8.4 g/dl	8.8 g/dl	10 g/dl
Lowest Hb (mg/dl)		4 g/dl	6 g/dl	7 g/dl	6.3 g/dl	7.3 g/dl	2.3 g/dl	7.3 g/dl	5.6 g/dl	6.2 g/dl	7 g/dl
Reticulocyte count (%)	0.5%–2.5% of RBCs	0.4	0.3	0.3	0.4	0.3	0.4	0.4	0.3	0.4	0.4
Leukopenia	WBC < 4000/mm^3^	No	No	No	Yes	No	Yes	Yes	No	No	No
Thrombocytopenia	<1 lakh/mm^3^	No	No	No	Yes	No	Yes	Yes	No	No	No
Bone marrow examination		Not done	Not done	Not done	Not done	Not done	Decreased erythroid lineage	Not done	Not done	Normal	Not done
Coinfection (CMV / BKV / EBV)		Nil	Nil	CMV	Nil	Nil	Nil	Nil	Nil	Nil	Nil
Treatment		RIS + IVIG	RIS + IVIG	RIS + IVIG	RIS	RIS	RIS + IVIG	RIS	RIS + IVIG	RIS + IVIG	RIS
Reduction of MMF		Yes	Yes	Yes	Yes	Yes	Yes	Yes	Yes	Yes	Yes
Stopped MMF		Yes	Yes	Yes	No	Yes	Yes	No	No	No	No
Transfusion		3 PINT	2 PINT	2 PINT	1 PINT	Not transfused	8 PINT	Nil	2 PINT	1 PINT	1 PINT
Time taken for sustained improvement in Hb post treatment		2 weeks	3 weeks	4 weeks	1 week	3 weeks	1 week	1 week	1 week	1 week	1 week
Graft dysfunction		No	No	No	No	Yes	Yes	No	No	No	Yes
Liver dysfunction		No	No	No	No	No	No	No	No	No	No
Outcomes at 6 months post-infection		Recovered	Recovered	Recovered	Recovered	Death due to cerebral abscess	Death due to multiple infections	Recovered	Recovered	Recovered	Recovered

F, female; M, male; LRRATX, live related renal allograft transplant; DDRATX, deceased donor renal allograft transplant; ATLG, anti-T lymphocyte globulin; RIS, reduction of immunosuppression; CMV, cytomegalovirus; PCR, polymerase chain reaction; IVIg, intravenous immunoglobulin; PCR, polymerase chain reaction.

**Definitions in the context of the table**

**Graft dysfunction (renal allograft dysfunction)**

● Decline in kidney function compared to baseline–typically detected as a rise in serum creatinine of ≥15% above baseline, which prompts further evaluation or biopsy when unexplained by reversible causes such as dehydration, drug toxicity, or obstruction.

**Liver dysfunction** is defined as clinically relevant impairment of hepatic function, as determined by:

● Serum alanine aminotransferase (ALT) or aspartate aminotransferase (AST) levels ≥ 2 times the upper limit of normal for the reference laboratory. And/or

● Elevated total bilirubin > 2 mg/dL. And/or

● Prolonged international normalized ratio (INR) > 1.5 in the absence of other causes (e.g., anticoagulation).

**Recovered is defined as:**

● Achievement of sustained hematological recovery: Hemoglobin normalization (≥12 g/dL for females, ≥13 g/dL for males, per cohort criteria) and resolution of reticulocytopenia (reticulocyte count > 1% of RBCs), confirmed by two consecutive measurements at least 1 week apart. And

● Stable renal allograft function: Creatinine level and eGFR return to pre-infection baseline; absence of ongoing graft dysfunction as defined above. And ∙ Normal liver enzymes.

Three patients (30%) developed concomitant leukopenia and thrombocytopenia. Bone marrow examination was performed in two patients; one demonstrated classical findings of decreased erythroid lineage with giant pronormoblasts containing viral inclusions, while the other had a normal marrow study. One patient exhibited co-infection with cytomegalovirus (CMV).

### Immunosuppression and transplant details

Allografts were ABO compatible. Induction immunosuppressive regimens included anti-T lymphocyte globulin (ATLG) at 3.5 mg per kg single dose in 7 patients, with the remainder receiving no induction. Maintenance immunosuppression consisted of tacrolimus (0.1 mg/kg twice daily), mycophenolate mofetil (MMF; 750 mg twice daily for 3 months post-transplant, followed by 500 mg twice daily), and prednisolone.

Two patients had documented allograft rejection episodes prior to parvovirus diagnosis, necessitating escalation of immunosuppression.

### Laboratory findings

Baseline hemoglobin ranged from 8.4 to 11 g/dL prior to infection, with nadir hemoglobin values observed between 2.3 and 7.3 g/dL (mean decrease 3.72 ± 1.22 g/dL). Peripheral blood smears consistently revealed normocytic normochromic anemia with reticulocytopenia (<1%). Leukopenia (WBC < 4000/μL) and thrombocytopenia (<150,000/μL) appeared simultaneously in 3 patients (30%).

### Treatment and therapeutic response

Initial management targeted reversible causes of anemia, including supplementation for iron, vitamin B12, and folate deficiencies. Reduction in immunosuppression (RIS), primarily via MMF dose reduction or discontinuation, was implemented in all patients (13).

Within 7–10 days of reduction of immunosuppression RIS, 4 patients (40%) exhibited a sustained upward trend in hemoglobin. The remaining 6 patients who failed to respond to reduction of immunosuppression at the end of 7 to days, received adjunct intravenous immunoglobulin (IVIg) at 400 mg/kg for five consecutive days. Among IVIg recipients, 4 achieved hematological recovery within 4 weeks and 2 patients at the end of 5 weeks.

Eight patients (80%) required packed red blood cell transfusions during the clinical course, with transfusion requirements ranging from 1 to 8 units.

Desidustat, a hypoxia-inducible factor prolyl hydroxylase inhibitor (HIF-PHI), was administered to all patients as adjunct therapy.

### Outcomes and complications

Median time to sustained hemoglobin improvement among responders was 2.14 weeks (range 1–4 weeks) following reduction of immunosuppression followed by use of fixed doses of IVIg of 400 mg/day for 5 days. At 3 months follow-up, 4 patients achieved full hematological and clinical recovery without graft dysfunction. At the end of 6 months, 8 patients attained full hematological recovery.

Four patients experienced graft dysfunction secondary to other causes: one due to slow graft function which improved to normal eventually, two developed chronic allograft dysfunction (one from recurrent infections, the other due to chronic antibody-mediated rejection), and one case was attributed to acute gastroenteritis which recovered completely once gastroenteritis subsided. Importantly, none of the graft dysfunctions were directly attributed to B19V infection, as confirmed by biopsy findings revealing acute tubular injury without viral cytopathic changes. Biopsy was performed with a minimum gap of 4 weeks after IVIG infusion.

There were two deaths during the follow-up period: one patient succumbed to cerebral abscess while another died due to multiple infections unrelated to B19V.

No patients exhibited fever, rash, neurological symptoms, myocarditis, or arthropathy during the course of infection.

### Statistical analysis

We evaluated the hematologic impact of B19V infection by comparing baseline hemoglobin (Hb) levels prior to infection and the nadir Hb values following infection within the same patient cohort (*n* = 10). The difference in Hb was assessed for normality using the Shapiro-Wilk test (*p* = 0.08), indicating no significant deviation from normality.

A paired *t*-test demonstrated a statistically significant reduction in Hb during infection (mean baseline Hb 9.62 ± 0.79 g/dL vs. nadir 5.90 ± 1.61 g/dL; *t*(9) = 6.82, *p* < 0.001), with a large effect size (Cohen’s d ≈ 2.16), confirming the clinical severity of anemia induced by B19V (Table 2).

**TABLE 2:** Summary table with key statistical results and effect size.

Variable	Test	Statistic	*P*-value	Effect size (r or d)	Interpretation
Baseline vs. lowest Hb	Paired *t*-test	6.82	<0.001	Cohen’s d ≈ 2.16 (large)	Significant decrease; large effect
Time from transplant to infection	Mann-Whitney U	3.0	0.23	r ≈ 0.34 (moderate)	Trend to longer time in deaths
RBC transfusions (units)	Mann-Whitney U	7.5	1.0	r ≈ 0.0 (negligible)	No difference detected
Time to hemoglobin improvement	Mann-Whitney U	7.0	0.88	r ≈ 0.0 (negligible)	No difference

To explore clinical factors associated with patient outcomes (recovery, *n* = 8 vs. death, *n* = 2), we compared transfusion requirements, time to Hb improvement, and time from transplantation to infection using Mann-Whitney U tests due to the small sample size and skewed distributions. No statistically significant differences were detected between the outcome groups for transfusion volume (*p* = 1.0), or time to Hb improvement (*p* = 0.88). However, there was a non-significant trend toward longer duration from transplant to infection in patients with fatal outcomes (median 359 weeks) compared to survivors (median 4.5 weeks) (*p* = 0.23).

Effect size calculations (r) for the Mann-Whitney tests revealed negligible effects for transfusions and time to Hb improvement, and a moderate effect size for time from transplantation to infection (r ≈ 0.34), suggesting potential clinical relevance despite statistical nonsignificance.

## Discussion

This study demonstrates that B19V infection is an important yet under-recognized cause of severe, refractory anemia in KTRs, with a prevalence of 8.5% in this cohort. This supports previous reports that cite B19V as a notable opportunistic viral pathogen in transplant recipients leading to pure red cell aplasia (1, 9).

Consistent with prior literature, all patients in this series presented with symptoms of fatigue and hypoproliferative anemia characterized by a markedly low reticulocyte count (<1%), reflecting the virus’s tropism for erythroid progenitors via the P antigen receptor. The absence of classical rash or arthropathy, commonly observed in immunocompetent hosts, underscores the often subtle clinical presentation in immunosuppressed patients. Furthermore, the concomitant occurrence of leukopenia and thrombocytopenia in some patients aligns with previous findings indicating broader hematopoietic suppression or bone marrow involvement in severe cases (3, 4).

The temporal profile in this study revealed a median onset of B19V infection approximately 6 weeks post-transplantation, highlighting the critical period of heightened immunosuppression during which patients are vulnerable to opportunistic infections. Notably, patients with late-onset infection (beyond 1 year) showed a trend toward worse outcomes, possibly attributable to cumulative immunosuppression or comorbidities (1, 6).

Management was centered on reducing immunosuppression to facilitate viral clearance, while carefully mitigating the risk of graft rejection. Initially MMF dose was reduced by 50%, in case of no improvement at the end to of 5–7 days, if was stopped. If there was no improvement even after stopping MMF, tacrolimus was changed to cyclosporine (14). If there was no improvement with reduction of immunosuppression at the end of 7 days - IVIg therapy was administered at 400 mg/kg/day for 5 days. Supportive measures, such as blood transfusions and use of Desidustat address the symptomatic burden of anemia. Serial monitoring of hemoglobin ensured timely response assessment and relapse detection.

However, 60% of patients required adjunctive intravenous immunoglobulin (IVIg) therapy due to inadequate response to reduction in immunosuppression alone. IVIg provides passive immunity and contains neutralizing antibodies against B19V, This protocol of reserving IVIg for resistant cases (those who do not respond to reduction of immunosuppression at the end of 7 days), is partly influenced by financial considerations, it parallels resource-conscious treatment approaches in low- and middle-income settings (7, 8, 15, 16).

Remarkably, the erythropoiesis-stimulating agent Desidustat, a hypoxia-inducible factor prolyl hydroxylase inhibitor (HIF-PHI) approved for anemia treatment in India, was employed in all patients as adjunct therapy. Desidustat stimulates endogenous erythropoietin production and may represent a valuable adjunctive therapy in viral anemia, although further prospective studies are required to establish efficacy in this context (8, 9).

The study reported an 80% transfusion rate during illness, underscoring the severity of anemia and the burden of supportive care. Importantly, the graft dysfunction observed in two patients was attributed to causes other than B19V infection, including acute rejection and infectious complications, supported by biopsy findings lacking viral cytopathic changes. This suggests that timely diagnosis and management of B19V can prevent direct viral nephropathy or graft loss (9, 17).

Despite two patient deaths related to severe infections unrelated to B19V, the majority achieved hematologic recovery within a median of 2 weeks after initiation of therapy (RIS ± IVIg). These findings align with global data highlighting favorable outcomes with early intervention (9).

Patients experiencing poor outcomes, including death or severe morbidity, tended to have several common features. Infection diagnosed late after transplantation (beyond 200 weeks) was associated with worse prognosis, potentially due to cumulative immunosuppressive burden or patient frailty. Those with significant graft dysfunction–such as chronic antibody-mediated rejection or chronic allograft deterioration–fared worse and had more complications. Additionally, more severe anemia, reflected by very low nadir hemoglobin (e.g., 2.3 g/dL), and higher volumes of red blood cell transfusions correlated with poorer outcomes. Coinfections, including cytomegalovirus or other serious infections like cerebral abscesses, also contributed to mortality. Patients who failed to respond promptly to reduction in immunosuppression or intravenous immunoglobulin therapy showed unfavorable clinical courses. Finally, suboptimal immunosuppression management, particularly in those with prior rejection episodes requiring increased immunosuppression, may predispose individuals to infections and adverse outcomes.

This study reinforces the importance of maintaining a high index of suspicion for B19V infection in KTRs exhibiting unexplained, refractory anemia, particularly within the early post-transplant period. Routine surveillance may not be warranted; however, targeted PCR testing in cases of persistent anemia can facilitate prompt diagnosis and intervention (18, 19).

There is ongoing debate regarding the optimal management of anemia in post-transplant patients, particularly surrounding the use of erythropoietin (EPO) and newer hypoxia-inducible factor prolyl-hydroxylase inhibitors (HIF-PHIs). EPO remains central to anemia correction, and its administration has been shown to improve graft survival and reduce all-cause mortality in transplant recipients. However, concerns persist about potential adverse cardiovascular events, risk of excessive hemoglobin correction, and EPO resistance in certain patients (20).

Hypoxia-inducible factor prolyl-hydroxylase inhibitors represent a novel class of oral agents that stimulate endogenous EPO production and promote iron metabolism through hepcidin reduction. Recent meta-analyses and clinical trials show that HIF-PHIs can effectively raise hemoglobin and may serve as alternatives to ESAs (erythropoiesis-stimulating agents), with comparable efficacy and safety in CKD and post-transplant populations. Nonetheless, the long-term safety profile of these agents and their place in therapy remain under investigation, especially in transplant recipients. Personalized selection and careful monitoring of anemia therapies are recommended until more robust evidence clarifies their risks and benefits in this unique population (21–24).

Limitations include the small sample size and single-center design, which may limit generalizability. Longer observation (12–24 months) would be valuable to confirm the durability of hematologic recovery, graft stability, and viral suppression. Serial quantitative B19V antibody titers were not measured. These could help determine whether IVIg facilitated endogenous immune response. Future multicenter studies with larger cohorts are warranted to validate risk factors, optimize treatment protocols, and evaluate long-term outcomes.

## Conclusion

Parvovirus B19 infection is an important and under-recognized cause of severe, refractory anemia in kidney transplant recipients, particularly in the early post-transplant period. Early diagnosis using polymerase chain reaction (PCR) and targeted testing in patients with persistent anemia are crucial for timely intervention. Reduction in immunosuppression, with or without adjunctive intravenous immunoglobulin therapy, constitutes an effective treatment strategy that can lead to favorable hematologic recovery. The use of erythropoiesis-stimulating agents such as Desidustat may offer additional benefit in persistent cases. Routine surveillance for B19V infection in all transplant recipients may not be justified; however, a high index of suspicion in those with unexplained anemia is essential to improve outcomes, prevent graft dysfunction, and reduce morbidity. Larger multicenter studies are warranted to further define optimal diagnostic and therapeutic approaches for this rare but clinically significant infection in the Indian transplant population.

## Data Availability

The data analyzed in this study is subject to the following licenses/restrictions: Datasets can be obtained from the author on reasonable request. Datasets are not publicly available due to privacy concerns. Requests to access these datasets should be directed to mythri.nish@gmail.com.
